# Paediatric HIV treatment failure: a silent epidemic

**DOI:** 10.7448/IAS.18.1.20090

**Published:** 2015-07-23

**Authors:** Jonathan M Bernheimer, Gem Patten, Thembisa Makeleni, Nompumelelo Mantangana, Nombasa Dumile, Eric Goemaere, Vivian Cox

**Affiliations:** Medecins Sans Frontieres, Khayelitsha, South Africa

**Keywords:** HIV, treatment failure, Khayelitsha, paediatrics, viral load, primary care

## Abstract

Paediatric antiretroviral treatment (ART) failure is an under-recognized issue that receives inadequate attention in the field of paediatrics and within HIV treatment programmes. With paediatric ART failure rates ranging from 19.3% to over 32% in resource limited settings, a comprehensive evaluation of the causes of failure along with approaches to address barriers to treatment adherence are urgently needed.

In partnership with the local Department of Health, a pilot programme has been established by Medecins Sans Frontieres (MSF) in Khayelitsha, South Africa, to identify and support paediatric HIV patients with high viral loads and potential treatment failure. Through detailed clinical and psychosocial evaluations and adherence support with an innovative counselling model, treatment barriers are identified and addressed.

Demographic and clinical characteristics from the cohort show a delayed median start date for ART, prolonged viraemia including a large number of patients who have never achieved viral load (VL) suppression, a low rate of regimen changes despite failure, and a high percentage of pre-adolescent and adolescent patients who have not gone through the disclosure process.

Stemming this epidemic of paediatric treatment failure requires programmatic responses to high viral loads in children, starting with improved “case finding” of previously undiagnosed HIV-infected children and adolescents. Viral load testing needs to be prioritized over CD4 count monitoring, and flagging systems to identify high VL results should be developed in clinics. Clinicians must understand that successful treatment begins with good adherence, and that simple adherence support strategies can often dramatically improve adherence. Moreover, appropriate adherence counselling should begin not when the child fails to respond to treatment. Establishing good adherence from the beginning of treatment, and supporting ongoing adherence during the milestones in these children's lives is key to sustaining treatment success in this vulnerable HIV-infected patient population.

While great progress has been made in the last 15 years in the field of paediatric HIV diagnosis and treatment, a growing problem has emerged that threatens to undermine much of the work that has been done. Paediatric antiretroviral treatment (ART) failure is an under-recognized issue that receives inadequate attention in the field of paediatrics and within HIV treatment programmes. This is in part due to a general absence of viral load (VL) testing in countries with a high burden of disease and limited resources, as well as the long-held belief that immunologic immaturity in children prohibits or prolongs VL suppression. While few studies have fully evaluated cohorts of children with virologic failure, rates of failure in the paediatric age group are reported to range from 19.3% to over 32% in resource limited settings [1,2]. We describe below the characteristics of children enrolled in a pilot programme to support children failing ART at two well-established primary care clinics in Khayelitsha, South Africa.

In Khayelitsha, there is a high HIV burden (37% antenatal prevalence, 2011) [3] and estimated good ART coverage. The ART programme was established in 2001 by Médecins Sans Frontières (MSF) and government health services and comprise 11 primary care clinics that are largely nurse managed [4–7]. Current South African Department of Health paediatric HIV guidelines recommend that clinicians consider a regimen change if the patient has experienced virologic failure – defined as two consecutive VLs >1000 copies/mL – despite a good adherence record. Without further specific guidance, clinicians are often uncertain how to assess adherence of HIV-infected children and their caregivers, as well as how to provide structured adherence support at the time of treatment failure. This hesitancy can lead to delays in switching regimens, the accumulation of resistance mutations, and potential long-term neurodevelopmental sequelae.

In July 2013, MSF partnered with the Department of Health to develop a pilot programme to identify and support paediatric and adolescent patients with high VLs. The programme consists of a comprehensive evaluation of each patient's medical and psychosocial history, HIV and general paediatric clinical care, structured adherence support through counsellor facilitated support groups, combined clinical/adherence clinician consultations, and home visits when possible by community care workers. Patients aged 0–19 years are eligible for enrolment if their previous VL is >1000 RNA copies/ml or their previous two VLs are >400 RNA copies/ml. Patients come for a series of visits (mostly once a month) and attend group and individual support sessions at each visit. Table 1 shows routine data on gender, age, treatment history, virologic history, and HIV disclosure status obtained upon enrolment.

**Table 1:** Demographic and clinical characteristics of HIV-infected paediatric patients attending the treatment failure programme in Khayelitsha, South Africa, in 2014

Number of patients who qualified for the programme out of the total paediatric patient population	140/465	30%
Number (percent) of patients enrolled in programme	118	84%
Number (percent) of females	52	44%
Median age (IQR)	10 years	5–14
Median age at ART start (IQR)	3.4 years	0.6–7.2
Median time on ART at enrolment (IQR)	3.9 years	2.5–7.5
Median viral load in copies/ml on enrolment (IQR)	13,141	2529–55,130
Number (percent) who never achieved virologic suppression prior to enrolment	39	33%
Number (percent) of patients on a non-nucleoside (NNRTI) regimen at enrolment	36	30%
Number (percent) of patients on a protease inhibitor (PI) regimen at enrolment	73	62%
Number (percent) of patients on 3TC monotherapy at enrolment	5	4%
Number (percent) of patients not on ART at enrolment	4	3%
Number (percent) of patients with a regimen change prior to enrolment	24	20%
Median CD4 count in cells/µl at enrolment (IQR)	687	(449–1164)
Number (percent) of patients aged 10–15 years old fully disclosed to at enrolment	48	80%

High rates of paediatric virologic failure were observed in a well-established primary care HIV setting, in line with data obtained in previous studies [1,2]. The median age of starting ART in this predominantly vertically infected cohort was 3.4 years, indicating a need for earlier identification of HIV-infected children and possible missed opportunities for diagnosis. Furthermore, one third of patients had never achieved virologic suppression since starting treatment. This prolonged viraemia could be explained by inadequate systems in clinics to locate failing children or a lack of knowledge or comfort by clinicians to address high VLs; this is also mirrored in the percentage of children (80%) that were not switched despite prolonged failure. Twenty percent of patients aged 10–15 years had not been fully disclosed to upon enrolment, known to be a risk factor for failure.

To stem this epidemic of paediatric treatment failure, programmatic responses to high VLs in children need to be strengthened. First and foremost, improved “case finding” of previously undiagnosed HIV-infected children is urgently needed to prevent the long-term sequelae of untreated HIV infection in young children. Second, VL testing of children should be prioritized over CD4 count monitoring in settings with limited resources; VL monitoring reduces the time failing treatment, increases appropriate switching to second-line ART, and minimizes immunologic decline. To this end, simple flagging systems for high VLs can assist clinics to identify those patients with high VL results and can be instituted with minimal resources in every HIV clinic that treats children. Third, clinicians need to understand that the most important factor in good paediatric HIV care begins with achieving good adherence. Often it is simple adherence support strategies that clinicians can use in busy clinics that greatly improve the quality of patient support that children and their caregivers receive. Lastly, adherence support should not start when the child has a high VL. Appropriate counselling should begin during the process of ART initiation and continue to be re-assessed as the child reaches developmental milestones. Too often, proper basic adherence counselling is only provided once the child has begun failing treatment. Periodic ongoing counselling is essential with paediatric patients since their psychosocial situations frequently changes and new adherence barriers often arise.

In summary, one third of children aged 0–19 years were failing antiretroviral therapy in two longstanding primary care HIV clinics, with 33% never achieving viral suppression after initiation of ART. Only by addressing the core deficiencies in paediatric HIV care – insufficient early diagnosis of HIV-infected children, lack of VL monitoring and clinician comfort in how to respond to high VLs, and unstructured and inadequate adherence counselling – will we begin to achieve durable VL suppression in the paediatric HIV population and curb this silent epidemic.
